# Discovering common pathogenic processes between COVID-19 and HFRS by integrating RNA-seq differential expression analysis with machine learning

**DOI:** 10.3389/fmicb.2023.1175844

**Published:** 2023-05-05

**Authors:** Fatima Noor, Usman Ali Ashfaq, Abu Bakar, Waqar ul Haq, Khaled S. Allemailem, Basmah F. Alharbi, Wafa Abdullah I. Al-Megrin, Muhammad Tahir ul Qamar

**Affiliations:** ^1^Integrative Omics and Molecular Modeling Laboratory, Department of Bioinformatics and Biotechnology, Government College University, Faisalabad, Pakistan; ^2^Centre of Agricultural Biochemistry and Biotechnology (CABB), University of Agriculture, Faisalabad, Pakistan; ^3^Department of Medical Laboratories, College of Applied Medical Sciences, Qassim University, Buraydah, Saudi Arabia; ^4^Department of Basic Health Science, College of Applied Medical Sciences, Qassim University, Buraydah, Saudi Arabia; ^5^Department of Biology, College of Science, Princess Nourah bint Abdulrahman University, Riyadh, Saudi Arabia

**Keywords:** hemorrhagic fever with renal syndrome, COVID-19, transcriptomic data, hub genes, genes ontologies, pathways, machine learning

## Abstract

Zoonotic virus spillover in human hosts including outbreaks of Hantavirus and severe acute respiratory syndrome coronavirus 2 (SARS-CoV-2) imposes a serious impact on the quality of life of patients. Recent studies provide a shred of evidence that patients with Hantavirus-caused hemorrhagic fever with renal syndrome (HFRS) are at risk of contracting SARS-CoV-2. Both RNA viruses shared a higher degree of clinical features similarity including dry cough, high fever, shortness of breath, and certain reported cases with multiple organ failure. However, there is currently no validated treatment option to tackle this global concern. This study is attributed to the identification of common genes and perturbed pathways by combining differential expression analysis with bioinformatics and machine learning approaches. Initially, the transcriptomic data of hantavirus-infected peripheral blood mononuclear cells (PBMCs) and SARS-CoV-2 infected PBMCs were analyzed through differential gene expression analysis for identification of common differentially expressed genes (DEGs). The functional annotation by enrichment analysis of common genes demonstrated immune and inflammatory response biological processes enriched by DEGs. The protein–protein interaction (PPI) network of DEGs was then constructed and six genes named RAD51, ALDH1A1, UBA52, CUL3, GADD45B, and CDKN1A were identified as the commonly dysregulated hub genes among HFRS and COVID-19. Later, the classification performance of these hub genes were evaluated using Random Forest (RF), Poisson Linear Discriminant Analysis (PLDA), Voom-based Nearest Shrunken Centroids (voomNSC), and Support Vector Machine (SVM) classifiers which demonstrated accuracy >70%, suggesting the biomarker potential of the hub genes. To our knowledge, this is the first study that unveiled biological processes and pathways commonly dysregulated in HFRS and COVID-19, which could be in the next future used for the design of personalized treatment to prevent the linked attacks of COVID-19 and HFRS.

## 1. Introduction

Hemorrhagic fever with renal syndrome (HFRS) is a major rodent-borne zoonosis caused by different species of hantaviruses including Hantaan virus (HTNV) (Lee et al., 1978), Puumala virus (PUUV) (Sironen et al., 2001), Seoul virus (SEOV) (Lee et al., 1982), and Dobrava-Belgrade virus (DOBV) (Papa, 2012). HFRS is primarily transmitted to humans through contact with rodents, excretions, or their saliva (Noor et al., 2022; Tariq and Kim, 2022). The disease can cause fever, hemorrhage, and kidney failure, and can be fatal in severe cases (Krautkrämer et al., 2013; Garanina et al., 2019). HFRS is a significant public health concern in some parts of the world, particularly in Asia and Europe. Most importantly, some latest studies suggested that individuals with HFRS are at increased risk of severe COVID-19 infection (Noor, 2020). COVID-19 is a highly infectious respiratory disease caused by the SARS-CoV-2 virus that was first identified in December 2019 (Li et al., 2020). This disease has spread rapidly around the world and has caused a global pandemic (Zhu and Cai, 2020). The symptoms of COVID-19 range from mild to severe, and the disease can be fatal, particularly in vulnerable populations such as the elderly and those with pre-existing health conditions (Flaherty et al., 2020). Additionally, the severity of COVID-19 appears to be influenced by various factors, including age, comorbidities, and viral load (Hasanoglu et al., 2021).

Recent studies reported that patients with HFRS who also had COVID-19 had more severe symptoms and longer hospital stays than those with HFRS alone (Singh et al., 2020; Subramaniam et al., 2022). Their study also reported a higher mortality rate among HFRS patients with COVID-19. Geladari et al. (2022) present a case study on patient with dialysis-dependent acute kidney injury due to hantavirus complicated with SARS-CoV-2 infection. Further, Cetin and Sahin (2021) presents a case followed up with the differential diagnosis of COVID-19 during the pandemic and diagnosed with HFRS due to hantavirus. To sum up, different case studies are reported in the literature, but, the absence of proper diagnostic tests hinders the diagnosis of co-infection among infected individuals. Overall, the evidence suggests a strong link between HFRS and COVID-19 comorbidity, particularly in terms of increased severity and mortality. However, the underlying mechanisms behind the increased severity of COVID-19 in individuals with HFRS are not yet fully understood. It is believed that the immune response to hantavirus infection may predispose individuals to a dysregulated immune response to SARS-CoV-2, leading to more severe illness (Wan et al., 2021). Furthermore, this co-infection leads to a more severe disease course, as both viruses can cause respiratory and renal failure, and ultimately their co-infection led to diagnostic challenges, as the symptoms of both diseases can overlap. The bell is ringing slightly thus, it is the need of the hour to develop effective diagnostic tools that can differentiate between single and co-infection and provide effective management for patients with COVID-19 and HFRS co-infection.

The spread of viral infections can be controlled through early detection of co-infections. However, current detection methods such as real-time polymerase chain reaction (RT-PCR) are not only time-consuming but also suffer from limited sensitivity and specificity (Bustin, 2000; Auwul et al., 2021). Moreover, the success of these techniques is highly reliant on skilled manpower, appropriate sample collection, and preparation, all of which pose significant challenges, particularly in developing countries. As a result, there is a pressing need for more efficient and reliable detection methods that can be easily deployed and implemented in resource-limited settings.

The transcriptomic analysis of COVID-19 and HFRS PBMCs can provide valuable insights in understanding the molecular mechanisms underlying the co-infection as well as the identification of potential biomarkers which could ultimately serve as potential targets for developing a single treatment strategy that could tackle both diseases simultaneously. Other studies have been carried out assessing other potential comorbidities with respect to COVID-19 including chronic kidney disease and diabetes mellitus (Auwul et al., 2021; Rahman et al., 2021). Thus, sensing these opportunities, this study combined transcriptomics and bioinformatics approaches for the identification of common genes and shared pathways among HFRS and COVID-19. Initially, the Differentially Expressed Genes (DEGs) were identified through transcriptomic analysis. The functional enrichment analysis was then performed to analyze the shared pathways for elucidating the immune response to the co-infection, which can lead to a better understanding of disease pathogenesis and potential targets for intervention. Later, a Protein–Protein interaction (PPI) network was constructed for the identification of hub genes from the pool of DEGs. However, the validity of these hub genes needs to be confirmed through rigorous validation processes. Thus, we employed supervised machine learning methods including Random Forest (RF), Poisson Linear Discriminant Analysis (PLDA), Voom-based Nearest Shrunken Centroids (voomNSC), and Support Vector Machine (SVM) to determine the validity of these hub genes. Here, for the first time, we have characterized the biological processes and pathways commonly dysregulated in COVID-19 and HFRS, which could be in the next future used in the designing personalized treatment of COVID-19 patients suffering from HFRS as comorbidity.

## 2. Materials and methods

### 2.1. Transcriptomic data acquisition

The collection of disease-related datasets is considered a preliminary step in the RNA-seq data analysis pipeline. The PBMCs transcriptomic datasets of SARS-CoV-2 (COVID-19) and HFRS-causing hantaviruses were collected from Gene Expression Omnibus (GEO) (Clough and Barrett, 2016), a public repository of functional high-throughput experimental data obtained through next-generation sequencing and microarrays. The criteria for disease-related dataset selection were completely based on the fact that all datasets must be from *Homo sapiens*. The dataset must contain transcriptomic data and the transcriptomic data contain no drug treatment. Two gene expression raw counts datasets of COVID-19 were retrieved through accession numbers; GSE160351 and GSE152418. The GSE160351 dataset was submitted by Brunetta et al. (2021) containing a total of 9 samples (three healthy controls and six infected individuals). While GSE152418 dataset was deposited by Arunachalam et al. (2020) comprising of total 34 samples (17 infected samples and 17 healthy samples). On the other hand, the other hantavirus-related dataset were obtained with accession number GSE158712 (Li et al., 2020) which consists of total of 30 samples (3 control and 27 infected samples) (Table 1). The raw sequence data of selected datasets were retrieved from the NCBI SRA toolkit using the prefetch command. The data was downloaded in .sra file which is not an acceptable format for different tools, therefore fastq-dump command was used for converting .sra data to .fastq format.

**Table 1:** Characteristics of datasets.

Disease	Acc. no#	Source	Total runs	Mock infected/control	Infected	Instrument
HFRS	GSE158712	PBMCs	30	3	27	Bgiseq-500 18
COVID-19	GSE160351	PBMCs	9	3	6	NextSeq 550
	GSE152418	PBMCs	33	17	16	Illumina NovaSeq 6,000

### 2.2. Data pre-processing

Depending on the sequencing technology, different strategies are used for processing and analyzing the raw sequencing data. Pre-processing the raw sequences data, such as performing quality control to check the read length, presence of any overrepresented sequences (k-mers), average quality score at each sequenced base, and percentage of GC content is now the most time-consuming step in RNA-seq data analysis. Firstly, the raw binary SRA data was turned into sequencing data. Later, the sequence data quality of each sample was controlled by FastQC (Brown et al., 2017). Further, to reduce the noise level, the obtained sequences were trimmed out for low-quality reads and adaptors by applying Sickle (Criscuolo and Brisse, 2013) Trimmomatic (Sewe et al., 2022), and FASTp (Sewe et al., 2022) tools on raw reads (Chen et al., 2018). Trimming of adapter sequences from raw reads was then performed using Sickle, Trimmomatic, and FASTp for identification of overlap adapters among forward and reverse reads. After trimming, the samples were prepared for further analysis.

### 2.3. Screening for differentially expressed transcripts and genes

After pre-processing, the raw reads were aligned with the reference genome to figure out which gene a read came from. Mapping and assembly of high-quality reads with reference genomes were performed with “New Tuxedo Suit” (HISAT2/StringTie) (Pertea et al., 2016), using default parameters. HISAT2 is a fast and accurate aligner for RNA-seq reads to a reference genome, and it uses a graph-based approach to account for splice junctions and other complex features in the genome. StringTie, on the other hand, is used for transcript assembly and quantification of gene expression. It takes the aligned RNA-seq reads produced by HISAT2 and assembles them into transcripts, estimates their abundances, and generates a file of gene expression values. Initially, indexing of the “*Homo sapiens*” reference genome and alignment of reads to the “*Homo sapiens*” reference genome was done using HISAT2. The aligned reads were then taken and used for the transcript assembly using the StringTie Tool. After assemblies, samples’ GTF (General Transfer Format) documents and Homo_sapiens.GRCh38.109.gtf. Were merged with the StringTie -merge option and transcript abundances of each sample were estimated with the StringTie-eB option (Goksuluk et al., 2019). Transcripts with variance across samples less than one were removed and then differentially expressed transcripts and genes between healthy individuals and infected individuals were screened using the stattest function from Ballgown [Version 2.12.0 (Weinstein et al., 2019)] with the getFC = TRUE parameter. The batch effect of two sources of transcriptome data was considered during our analysis. Transcripts and genes with a fold change >1.0 and value of *p* < 0.05 were identified as differentially expressed transcripts and genes. All DEGs, including the genes corresponding to the differentially expressed transcripts and differentially expressed genes screened out by Ballgown, were used for subsequent steps.

### 2.4. Identification of common transcriptional signatures and pathways between COVID-19 and HFRS PBMCs

Our study mainly aims to identify common transcriptional signatures, regulators, and pathways between COVID-19 and HFRS. At first, all the DEGs related to COVID-19 and HFRS were obtained and imported into a venn diagram tool^1^ to predict overlapped genes for uncovering their common pathogenic processes. Eventually, a group of mutual genes was acquired and considered for further analysis. The overlapped genes were then subjected to Clusterprofiler (Yu et al., 2012) and TopGO (Alexa and Rahnenführer, 2009) packages of R for the identification of common pathways shared among COVID-19 and HFRS. To identify significantly enriched GO terms and KEGG pathways in the analysis, a statistical threshold criterion was applied, with an adjusted *p*-value of less than 0.05. This criterion was used to select the most relevant and statistically significant GO terms and KEGG pathways. In short, the characterization of common transcriptional signatures, biological processes, molecular function, and pathways dysregulated in COVID-19 and HFRS, could be in the next future used for the design of personalized treatment of COVID-19 patients suffering from HFRS as comorbidity.

### 2.5. PPI network construction and identification of hub genes

Protein–Protein Interaction (PPI) networks are remarkably significant due to their high versatility, adaptability, and specificity. The functional interactions among overlapped genes with a combined score of more than 0.4 were determined using the Search Tool for the Retrieval of Interacting Genes/Proteins (STRING) database (von Mering et al., 2003). Initially, the overlapped genes between COVID-19 and HFRS were then submitted to the STRING database for the construction of the PPI network. The resulting PPI network was then subjected to Cytoscape version 3.8 (Shannon et al., 2003) for the identification of hub genes. Hub genes are the highly connected nodes that have a large number of interactions with other genes or proteins. These genes are considered critical components of the PPI network as they play important roles in maintaining the integrity and stability of the network, and they are often associated with key biological processes and pathways. Hub genes are identified by analyzing the topology of the PPI network, using measures such as degree, betweenness, or closeness centrality. In current study, degree methods available in CytoHubba was used for the identification of hub genes.

### 2.6. Performance evaluation of the hub genes with classification algorithms

**Figure 1:**
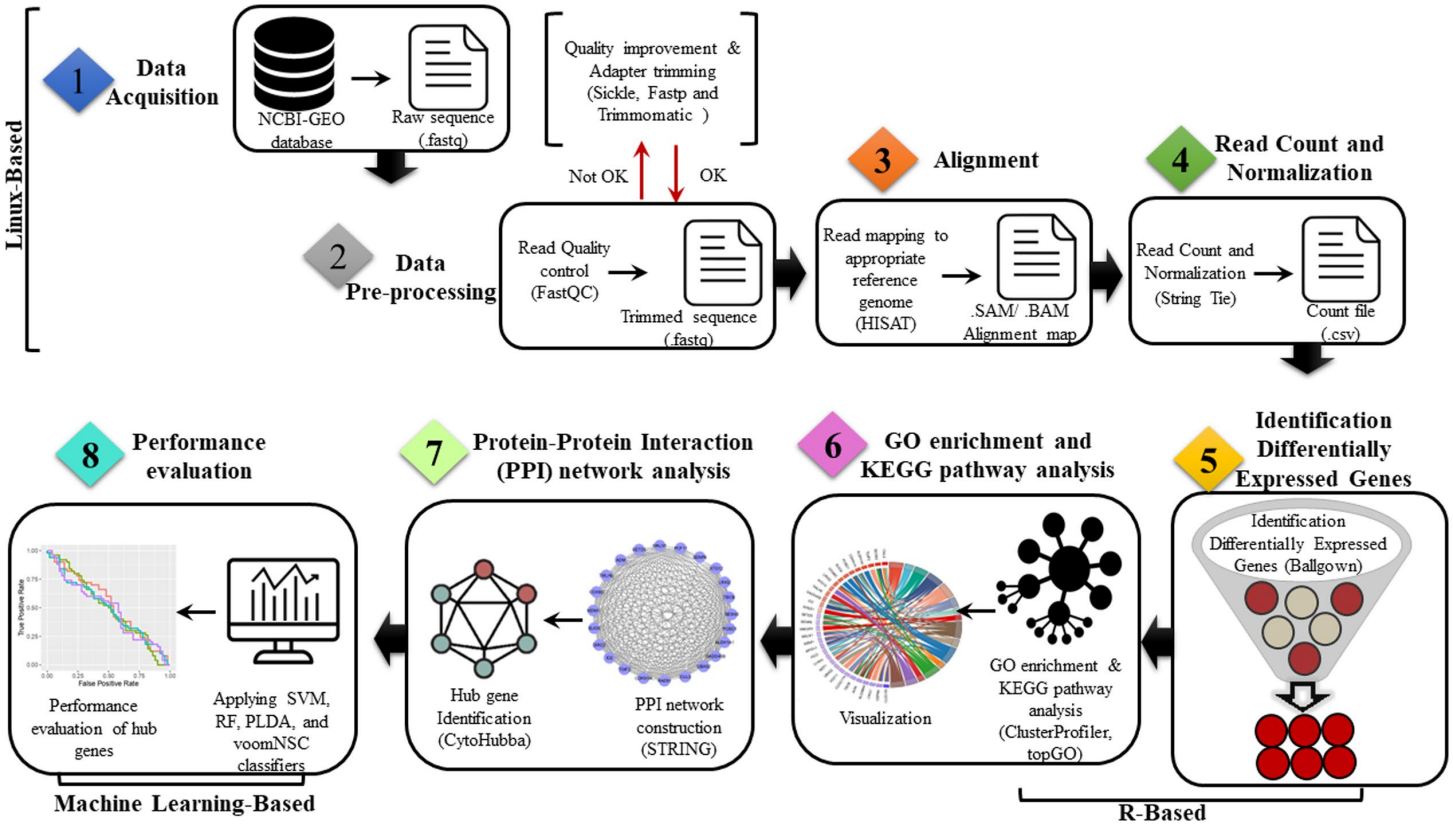
Proposed bioinformatics pipelines for the identification of common genes and dysregulated pathways in COVID-19 and HFRS.

To assess the credibility of the identified hub genes, five commonly used classification algorithms—support vector machine (SVM) (Boser et al., 1992) with radial basis kernel function, random forest (RF) (Ho, 1995), Poisson linear discriminant analysis (PLDA) (Witten, 2011), and Voom-based Nearest Shrunken Centroids (voomNSC)(Goksuluk et al., 2019) were employed. Datasets were split into two subsets, training data (70%) and testing data (30%). Then, the classification algorithms were conducted through the MLSeq package in R. The evaluation of model performance is typically subjective and based on a comparison of the model’s predictions with the known values of the dependent variable in a given dataset. However, for our study, we have defined the ideal model performance as having metrics’ results within the range of 70–90%. On the other hand, a performance exceeding 90–100% may indicate the possibility of overfitting. The DESeq normalization and VST transformation methods were applied to the count dataset for the SVM and RF classifiers. The HFRS dataset GSE158712 was utilized for the classification analysis, in which the hub genes were incorporated. The classification performance was evaluated using four metrics: accuracy, area under the receiver operating characteristic curve (AUC), sensitivity, and specificity based on the data with hub genes. An overview of the present study is shown in Figure 1.

## 3. Results

### 3.1. Screening of DEGs among COVID-19 and HFRS

**Figure 2:**
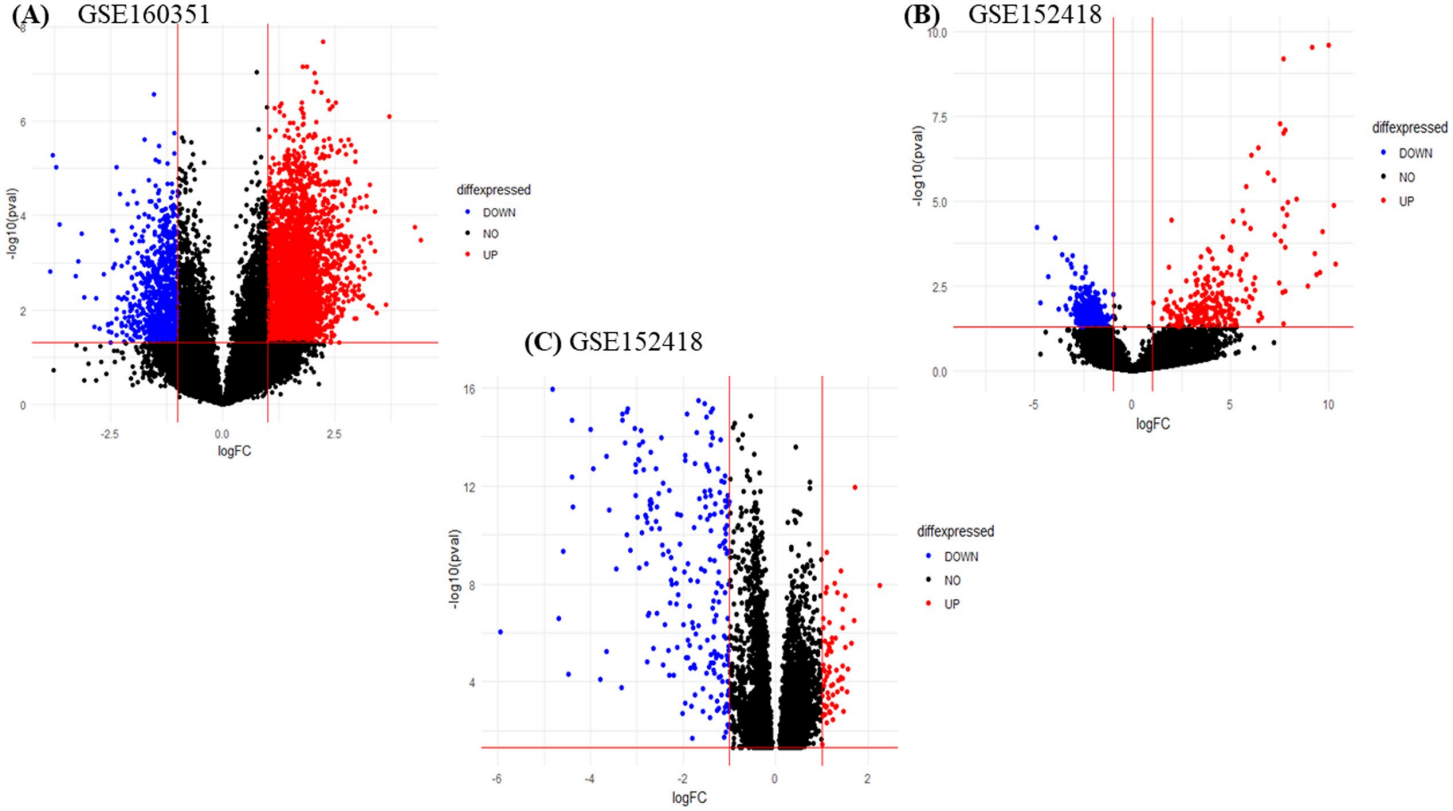
Volcano plot **(A)** GSE160351, **(B)** GSE152418, and **(C)** GSE152418.

We obtained two PBMCs gene expression datasets from COVID-19 infected subjects and matched healthy controls (with accession numbers, GSE160351 and GSE152418) for a total of 42 samples, 22 infected individuals and 20 healthy controls. The RNA-seq data analysis of GSE160351 and GSE152418 datasets yielded 1734 DEGs (1,108 upregulated and 626 downregulated). Screening criteria for identification of DEGs were set as value of *p* < 0.05 and LogFC >1.0. For GSE158712, the selection criteria for DEGs screening were set as value of *p* < 0.05 and LogFC >1.0. After the screening, 630 DEGs (390 downregulated and 240 upregulated) were identified from GSE158712. The volcano plot of DEGs obtained from GSE160351, GSE152418, and GSE158712 was shown in Figure 2 which provides a pictorial representation of upregulated DEGs, downregulated DEGs, and non-significant genes obtained from each dataset.

### 3.2. Identification of common transcriptional signatures between COVID-19 and HFRS PBMCs

**Figure 3:**
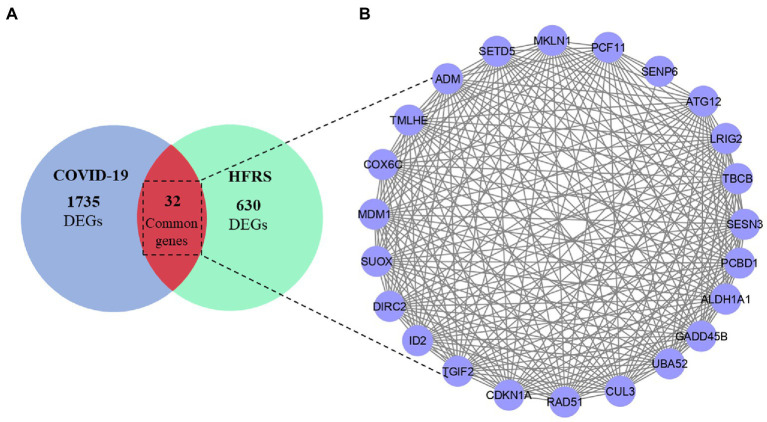
**(A)** Overlapped genes between COVID-19 and HFRS. **(B)** PPI network of 32 common dysregulated genes among COVID-19 and HFRS.

After DEGs identification, a Venn diagram was constructed which indicated 32 common genes between COVID-19 and HFRS. Among them, 17 genes were commonly upregulated and 15 DEGs were commonly downregulated in COVID-19 and HFRS. Overall, the comparative analysis of the transcriptional signatures characterizing COVID-19 and HFRS PBMCs suggests the presence of commonly dysregulated genes between COVID-19 and HFRS (Figure 3 and Supplementary Table S1). After the identification of common genes, a PPI network was constructed for analyzing interaction among 32 commonly dysregulated proteins.

### 3.3. Biological insights of the four-module genes

**Figure 4:**
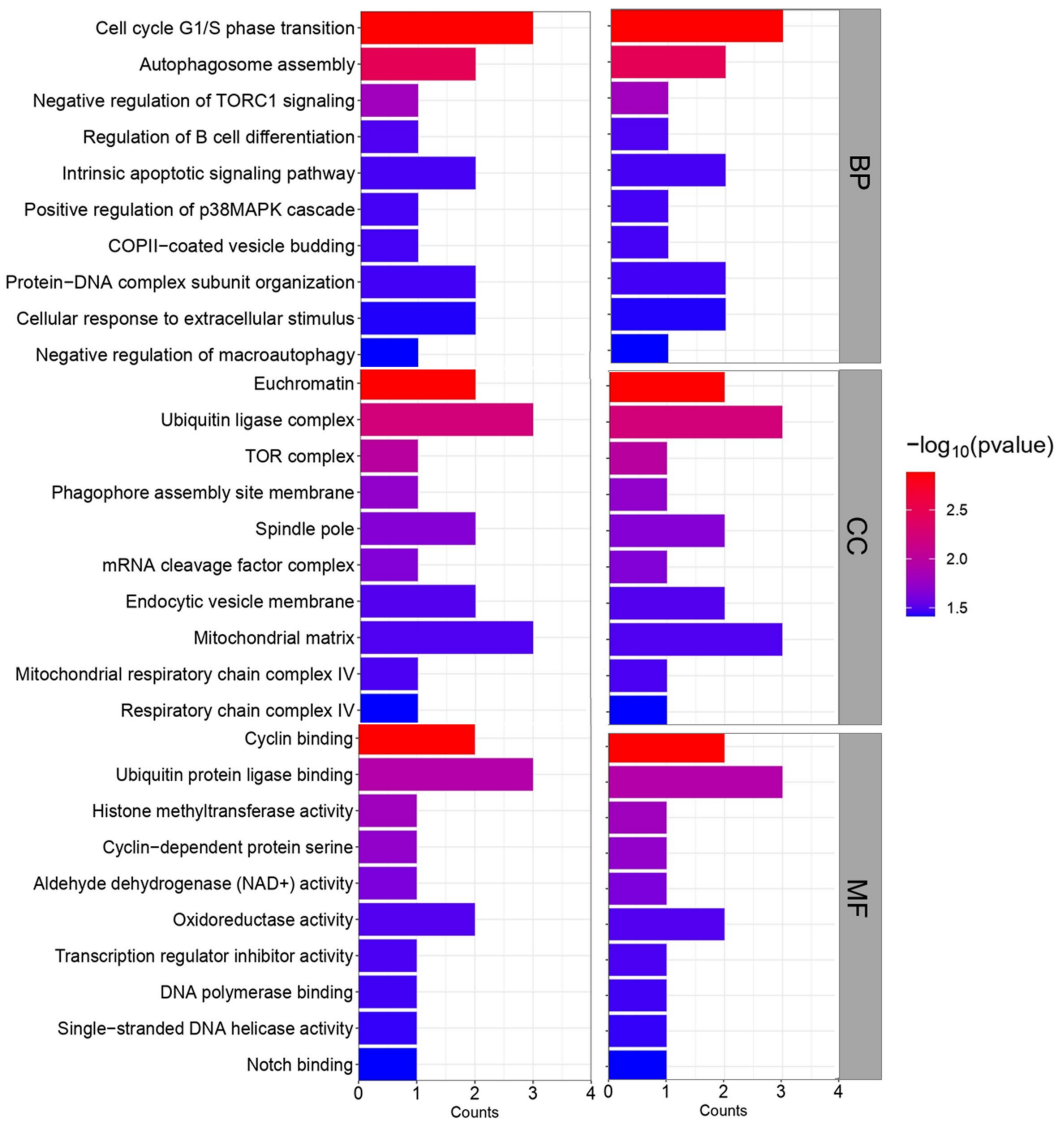
GO enrichment analysis of common 32 genes between COVID-19 and HFRS.

GO and KEGG pathway analysis was then performed to obtain further biological insight into the commonly dysregulated genes of HFRS and COVID-19. The findings of GO analysis revelated that in terms of BP, the genes were mainly involved in the intrinsic apoptotic signaling pathway, cell cycle G1/S phase transition, autophagosome assembly, negative regulation of TORC1 signaling, regulation of B cell differentiation, COPII-coated vesicle budding, positive regulation of p38MAPK cascade, protein-DNA complex subunit organization, cellular response to extracellular stimulus, and negative regulation of macroautophagy (Figure 4). The most significant cellular components (CC) overlapped genes are enriched in several cell compartments. The significant molecular function (MF) mainly enriched in the binding-related functions including cyclin binding, ubiquitin protein ligase binding, histone methyltransferase activity, cyclin-dependent protein serine, aldehyde dehydrogenase (NAD+) activity, oxidoreductase activity, transcription regulator inhibitor activity, DNA polymerase binding, single-stranded DNA helicase activity, and notch binding.

**Figure 5:**
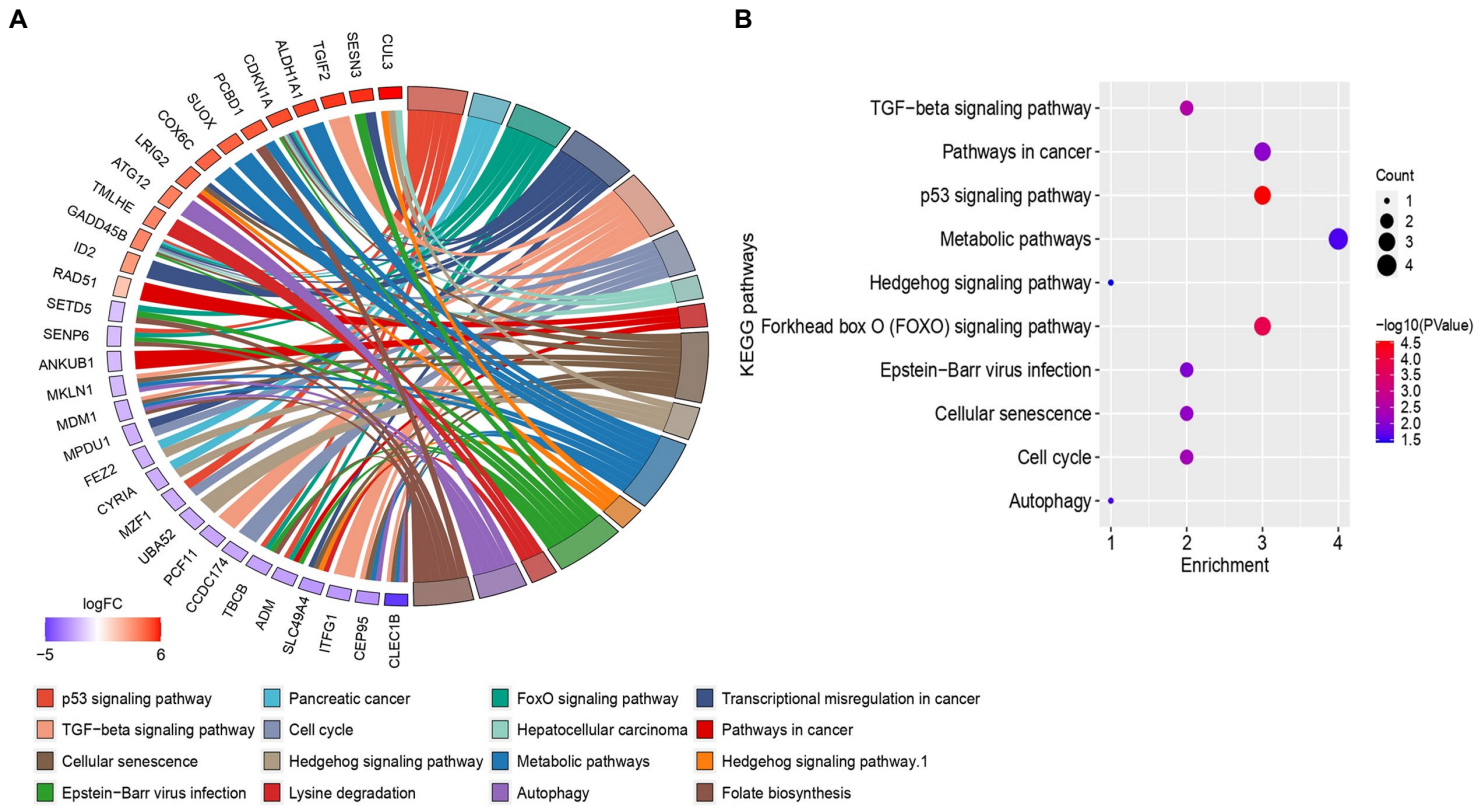
**(A)** Circos plots of closely related KEGG pathways and differentially expressed genes belonging to relevant pathways. **(B)** Bubble plot of top 10 significant KEGG pathways.

After GO enrichment analysis, KEGG pathway analysis was performed for providing biological context and insight into the underlying mechanisms of HFRS comorbidity in COVID-19 patients. Through clusterprofiler, only 16 KEGG pathways were identified which fulfill the criteria of value of *p* < 0.05, while those with value of *p* > 0.05 were excluded from the study. The KEGG pathways of overlapped genes are mainly enriched in several pathways such as infection-related pathways, i.e., epstein–barr virus infection, p53 signaling pathway, forkhead box O (FOXO) signaling pathway, TGF-beta signaling pathway, cell cycle cellular senescence, pathways in cancer, metabolic pathways, autophagy, and hedgehog signaling pathway. The circos plot represented the common genes along with their associated pathway are shown in Figure 5A. Gene involvement in the KEGG pathways was identified by colored connecting lines. Further, the bubble plot of top 10 significant KEGG pathways was presented in Figure 5B.

### 3.4. Identification of hub genes

**Figure 6:**
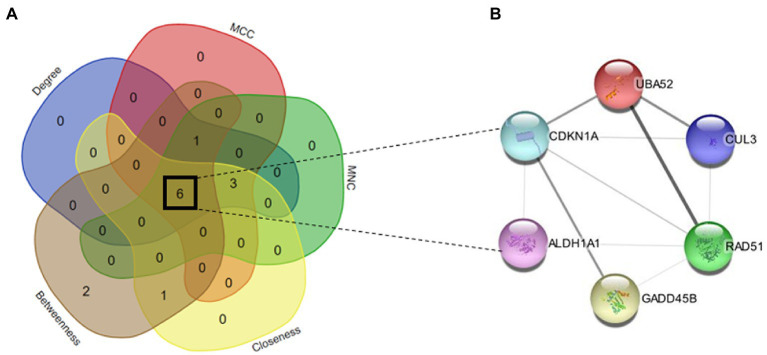
**(A)** Venn map of top hub genes of 5 algorithms (degree, MCC, MNC, closeness, and betweenness). **(B)** PPI network of six hub genes (GADD45B, UBA52, CDKN1A, RAD51, CUL3, and ALDH1A1).

The PPI network of common genes was constructed through the STRING database. The PPI network was then imported to Cytoscape for the identification of hub genes. There are 11 topological methods available in cytoHubba. From these methods, degree, MCC, MNC, closeness, and betweenness were selected for hub gene identification. The top 10 genes obtained from the degree, MCC, MNC, closeness, and betweenness were subjected to venn plot (Supplementary Table S2). A total of 6 six genes named GADD45B, UBA52, CDKN1A, RAD51, CUL3, and ALDH1A1 were found to be common in each method (Figure 6). The logFC values and *p*-values of selected hub genes are presented in Table 2.

**Table 2:** Summary of hub genes in COVID-19 and HFRS datasets.

Symbol	Gene name	HFRS datasets		COVID-19 datasets	
		log2FC (>1.0)	*P*-value (>0.05)	log2FC (>1.0)	*P*-value (>0.05)
RAD51	RAD51 recombinase	4.776201	0.009451	1.223918	3.50E-07
ALDH1A1	Aldehyde dehydrogenase 1 family member A1	4.29785	0.005062	3.74E-05	2.21E-06
UBA52	Ubiquitin A-52 residue ribosomal protein fusion product 1	3.572751	0.018842	1.071183	0.014559
CUL3	Cullin 3	−4.72487	0.010141	−1.32525	0.000372
GADD45B	Growth arrest and DNA damage inducible beta	−1.85687	0.022523	−1.82173	0.002635
CDKN1A	Cyclin dependent kinase inhibitor 1A	−1.90911	0.03701	−1.92171	0.001758

### 3.5. Performance evaluation of the hub genes with a classification algorithm

After the identification of hub genes, different supervised machine-learning classifiers were implemented to evaluate the discriminative performance of predicted hub genes. Four different types of popular classification algorithms including RF, SVM, PLDA, and voomNSC were executed for computing the performance measure including sensitivity, specificity, and accuracy of hub genes (Table 3). To execute this task, we divided the GSE158712 into test datasets and training datasets. MLSeq takes a matrix of raw counts as the input and performs normalization within-fold so that the normalization of the test fold is performed using coefficients estimated from the training folds. The process of randomly splitting samples into training/test folds, training the models, and then testing performance was repeated 10 times to obtain estimates of model performance. The performance metrics were averaged across the repeated folds. Accuracy explains the overall correctness of classification, and is defined as the proportion of all cases that are correctly classified or diagnosed. Mathematically, accuracy can be expressed as: Accuracy = (True Positives + True Negatives)/(True Positives + False Positives + True Negatives + False Negatives). Sensitivity, also known as recall or true positive rate, measures the proportion of actual positives that are correctly identified by the classification. Sensitivity = True Positives/(True Positives + False Negatives). Specificity, on the other hand, measures the proportion of actual negatives that are correctly identified by the classification or diagnostic test. Specificity = True Negatives/ (True Negatives + False Positives).

**Table 3:** Classification performance of six hub genes.

Classifiers	Accuracy (%)	Sensitivity (%)	Specificity (%)
Random forest (RF)	79.41%	88.24%	70.59%
Poisson linear discriminant analysis (PLDA)	70.59%	64.71%	76.47%
Voom-based Nearest Shrunken Centroids (voomNSC)	77.94%	88.24%	67.65%
Support vector machine (SVM)	76.47%	82.35%	70.59%

**Figure 7:**
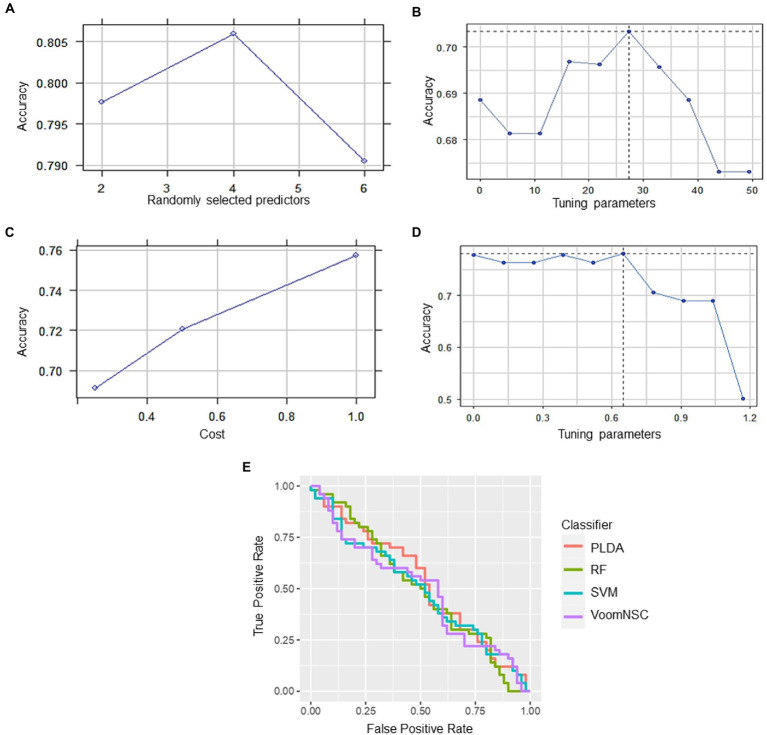
Classification performance of four different types of classifiers **(A)** RF, **(B)** PLDA, **(C)** SVM, and **(D)** voomNSC. **(E)** Receiver operating curve (ROC) plot of the four classifiers performance based on their accuracy, sensitivity, and specificity.

RF had the highest accuracy of 79.41%, indicating that it was able to correctly classify the samples with a high level of accuracy. The sensitivity of 88.24% suggests that the model was able to correctly identify most of the positive cases (i.e., samples with high expression of the hub genes), while the specificity of 70.59% indicates that it was less successful in correctly identifying the negative cases (i.e., samples with low expression of the hub genes). PLDA had a lower accuracy of 70.59% as compared to RF. The sensitivity of 64.71% suggests that the model was not able to correctly identify many of the positive cases, while the specificity of 76.47% indicates that it was better at identifying the negative cases compared to RF. On the other hand, voomNSC had an accuracy of 77.94%, indicating that it performed better than PLDA but not as well as RF. While the sensitivity was found to be 88.24% suggests that voomNSC correctly identify most of the positive cases, while the specificity of 67.65% indicates that it was less successful in correctly identifying the negative cases compared to both RF and PLDA. Finally, SVM had an accuracy of 76.47%, which is similar to VoomNSC. The sensitivity of 82.35% suggests that it was able to correctly identify most of the positive cases, while the specificity of 70.59% is similar to RF. Recently, Auwul et al. (2021) applied ML algorithms for the performance evaluation of hub genes. Their results indicated that SVM provides greater accuracy of 0.996 followed by RF: 0.955, PLDA: 0.821, and voomDLDA: 0.988. Similarly, we also observed satisfactory sample classification performance (accuracy >0.70) between affected and health control samples for both datasets. An accuracy value greater than 70% indicates great model performance The findings of the current study revealed that RF provides high accuracy of 79.41%as compared to SVM (76.47%), voomNSC (77.94%), and PLDA (70.59%) (Figure 7), suggesting the biomarker potential of the hub genes.

## 4. Discussion

HFRS is a potentially fatal infectious disease with worldwide distribution. PUUV, SEOV, DOBV, and HTNV are the primary causative agents of HFRS (Shid et al., 2022). PUUV, SEOV, DOBV, and HTNV are a member of rodent-borne viruses called hantaviruses that cause life-threatening human diseases in Europe and Asia. COVID-19 is a respiratory disease caused by the novel coronavirus SARS-CoV-2, which primarily affects the respiratory system (Yang et al., 2020). While HFRS and COVID-19 have distinct modes of transmission and clinical presentations, there have been reports of co-infections and comorbidities between the two diseases. But the reasons for this are not yet clear, but it is believed immune response to one disease may increase susceptibility or severity of the other such as kidney damage and compromised immune function, which may increase the severity of COVID-19. However, further research is needed to fully understand the nature of this relationship and to develop appropriate treatment strategies.

The presence of comorbidities in individuals co-infected with HFRS and COVID-19 represents a significant challenge that necessitates a solution. It is imperative to develop effective strategies to manage the comorbidities to improve clinical outcomes and reduce the burden of these diseases. Therefore, it is essential to investigate the fundamental genes and pathways involved in coninfection in order to unravel the molecular associations and mechanisms that are shared by these pathologies. To achieve this, the application of whole-genome transcriptomic analyses has been extensively utilized by researchers to explore autoimmune diseases, cancer, and neurodegenerative disorders, as well as to identify potential pathogenetic mechanisms and novel therapeutic targets. In the current study, integrative bioinformatics approaches were used for the comprehensive analysis of peripheral blood mononuclear cell (PBMC) transcriptomic changes occurring in HFRS and COVID-19. Our study uncovered six genes named RAD51, ALDH1A1, UBA52, CUL3, GADD45B, and CDKN1A were found to be commonly dysregulated among HFRS and COVID-19. Further, machine learning classifiers have gained immense popularity in various bioinformatics tasks. Therefore, we employed different machine learning classification algorithms on hub genes data to determine their efficacy. Our findings indicate that the classifiers performed satisfactorily with an accuracy of >0.70 for the classification of samples between COVID-19 and HFRS.

Auwul et al. (2021) applied the similar approach to identify the potential drug targets and pathways in COVID-19. Their findings proposed that PLK1, AURKB, AURKA, CDK1, CDC20, KIF11, CCNB1, KIF2C, DTL, and CDC6 were mainly enriched in the inflammatory and immune response, suggesting that these genes are significantly associated with viral infectious diseases. Similarly, Rahman et al. (2021) discovered common pathogenetic processes between COVID-19 and diabetes mellitus by differential gene expression pattern analysis. Their study, for the first time, characterized the biological processes and pathways commonly dysregulated in COVID-19 and diabetes mellitus, which could be in the next future used for the design of personalized treatment of COVID-19 patients suffering from diabetes mellitus as comorbidity. Their study proposed that SARS-CoV-2 could directly determine an impairment of insulin secretion, with consequent disruption of the metabolic control in people already suffering from diabetes mellitus or leading to the development of new-onset diabetes mellitus. Our study explores the possible risk of HFRS after COVID-19 infection by investigating the common molecular mechanisms. By taking advantage of the holistic viewpoint of systems biology, we were able to consider every aspect of both diseases and infer novel hypotheses. Further supplementary studies need to be conducted to clarify the association between COVID-19 and HFRS, as, at the moment, there is little known regarding both of these disease entities. Overall, our analysis highlights various infection-related pathways, i.e., epstein–barr virus infection, p53 signaling pathway, FOXO signaling pathway, TGF-beta signaling pathway, cell cycle cellular senescence, pathways in cancer, metabolic pathways, autophagy, and hedgehog signaling pathway which might be the potential links between both COVID-19 and HFRS.

Among six hub genes, RAD51 is a recombinase protein that plays a key role in homologous recombination (Huang et al., 2012). Previous studies demonstrated a potential association between RAD51 and COVID-19. It has been suggested that COVID-19 infection may interfere with the expression and activity of RAD51, thereby impairing DNA repair and increasing the risk of genetic mutations (Biering et al., 2021). Furthermore, studies have also shown that RAD51 may be involved in the inflammatory response to COVID-19 infection (Morenikeji et al., 2021). Elevated levels of inflammation are associated with severe COVID-19, and RAD51 has been shown to modulate inflammatory signaling pathways. In the same vein, RAD51 might play a role in the virus-host interaction by reducing viral replication during hantavirus infection. All these points strengthened the findings that dysregulation of RAD51 expression and activity may contribute to the development of severe HFRS. In conclusion, further research is needed to fully elucidate the mechanisms of RAD51 for understanding the common pathogenic processes between COVID-19 and HFRS, which could pave the way for the development of novel therapeutic strategies. On the other hand, ALDH1A1 has been implicated in the pathogenesis of the disease through its potential involvement in modulating the immune response and influencing the balance between pro-inflammatory and anti-inflammatory cytokines. Furthermore, CDKN1A, also known as p21, is a significant regulator of the cell cycle by controlling the activity of cyclin-dependent kinases (CDKs). In COVID-19, studies have reported that CDKN1A is upregulated in the lung tissue of infected patients, which could lead to the inhibition of virus replication and decreased inflammation in the host. CDKN1A has been shown to inhibit the replication of some viruses, including herpes simplex virus (HSV) and human immunodeficiency virus (HIV), by blocking the cell cycle progression of infected cells. In HFRS, studies have shown that CDKN1A is upregulated in the kidney tissue of HFRS patients, which may contribute to the development of renal injury (D’Souza, 2022). CDKN1A has been suggested to be involved in the regulation of cellular senescence and apoptosis, which are important processes in the development of renal injury in HFRS. Thus, more future studies are needed to fully understand the role of CDKN1A as well as other hub genes in both COVID-19 and HFRS.

There is limited information available on the translational activity of UBA52 and CUL3 specifically in HFRS. However, both UBA52 and CUL3 are involved in the ubiquitin-proteasome system, which plays an important role in the regulation of various cellular processes, including protein degradation and immune response (Meyer et al., 2020; Jiang et al., 2022). In general, dysregulation of the ubiquitin-proteasome system has been implicated in the pathogenesis of viral infections, including COVID-19, by influencing viral replication and modulating the host immune response (Seyoum, 2023). Therefore, it is possible that UBA52 and CUL3 may also play a role in the pathogenesis of COVID-19 and HFRS coinfection through their involvement in the ubiquitin-proteasome system. In short, there is no direct evidence of interactions between these six hub genes in the context of COVID-19 and HFRS, their involvement in processes related to DNA damage response, inflammation, and immune regulation suggests that they may be part of a larger interactome in their coinfection.

Further, GO and KEGG pathway analysis revealed that the commonly dysregulated genes are mainly involved in infection-related pathways, i.e., epstein–barr virus infection, p53 signaling pathway, forkhead box O (FOXO) signaling pathway, TGF-beta signaling pathway, cell cycle, cellular senescence, pathways in cancer, metabolic pathways, autophagy, and hedgehog signaling pathway. These pathways are known to play important roles in various cellular processes, such as cell cycle regulation, DNA damage response, cell proliferation, differentiation, and survival, and are often dysregulated in diseases such as cancer and viral infections. For instance, targeting genes involved in the FOXO signaling pathway may be helpful for treating COVID-19 and HFRS coinfection because it regulates immune responses, oxidative stress, and inflammation. For example, activation of FOXO3a has been shown to enhance the antiviral response by increasing the expression of interferon-stimulated genes and inhibiting viral replication (Wang et al., 2017). Additionally, FOXO3a regulates oxidative stress by increasing the expression of antioxidant enzymes, such as catalase and superoxide dismutase, and inhibiting reactive oxygen species production (Higuchi et al., 2013). In COVID-19 and HFRS, the immune response can be dysregulated, leading to a cytokine storm and inflammation. Activation of FOXO3a can suppress pro-inflammatory cytokines and reduce inflammation, thereby preventing tissue damage and improving clinical outcomes. Moreover, FOXO3a has been shown to promote autophagy which helps in clearing viral infections by degrading and recycling viral components (Wan et al., 2022). Therefore, the identification of genes involved in the FOXO signaling pathway may provide a novel therapeutic approach for COVID-19 and HFRS by regulating immune responses, oxidative stress, and inflammation. Overall, these common pathways may help to better understand the pathogenesis of HFRS and COVID-19, and targeting the linked genes could help in the development of new therapies to fight against the coinfections. As this field continues to evolve, future studies may benefit from incorporating network-based approaches to predict host-pathogen interactions, Therefore, future studies could incorporate the host-pathogen interactome to gain a deeper understanding of the disease mechanisms and identify additional potential therapeutic targets. For instance, Basu et al. (2022) reported the potential of network-based approaches to predict host-pathogen interactions and identify key host factors involved in viral infections. This approach could be a valuable addition to our computational pipeline in future studies.

To sum up, the integration of transcriptomic with bioinformatics approaches uncovered several pathways and common genes that are involved in both HFRS and COVID-19, suggesting potential similarities in their underlying pathophysiological mechanisms. The identified pathways and genes may serve as potential targets for the development of new therapeutics and which may lend a helping hand in understanding the pathogenesis of both diseases. However, it is important to note that RNA-seq analysis is just one tool in the development of potential therapies for comorbid HFRS and COVID-19. Other factors, such as clinical trials, animal studies, and epidemiological data, also need to be considered before any therapies can be developed and implemented. Additionally, the differences and similarities between HFRS and COVID-19 in terms of genetic factors, clinical manifestations, and disease progression need to be further investigated to develop targeted and effective treatments.

## 5. Conclusion

Comorbidities are associated with a higher risk of developing severe forms of COVID-19, with a consequent need for mechanical ventilation and an increased death rate. Increased severity of COVID-19 has been observed in patients with HFRS. Following that, the present research aimed to identify gene expression patterns and molecular pathways that were shared between COVID-19 and HFRS. A total of 32 genes were found to be common among COVID-19 and HFRS. From these 32 genes, six named RAD51, ALDH1A1, UBA52, CUL3, GADD45B, and CDKN1A were identified as the hub genes. Later, we demonstrated the classification performance of hub genes with an accuracy greater than 0.70 suggesting the biomarker potential of the hub genes. Further, our study uncovered immune-related pathways that were commonly dysregulated in PBMCs of both COVID-19 and HFRS. In summary, this study provides valuable insights into the molecular mechanisms of HFRS and COVID-19, which may lead to potential therapies for comorbidities. We propose that these events may have important roles in the onset or progression of HFRS. To sum up, our analysis describes possible mechanisms linking COVID-19 and HFRS, elucidating some unknown clues in between. Nonetheless, as this is a thorough computational work, further case reports and follow-up experiments of COVID-19 patients can corroborate these links.

## Data availability statement

The original contributions presented in the study are included in the article/Supplementary material, further inquiries can be directed to the corresponding author.

## Author contributions

FN collected the data, performed experiments, and write down the first draft of the manuscript. UAA, AB, WQ, KSA, BFA and WAM visualized and validated the results and revised the manuscript. KSA, BFA, WAM and MTQ arranged resources and funding for this study. UAA and MTQ designed and supervised this study, and finalized the manuscript. All authors approved the final version for submission.

## Funding

Princess Nourah bint Abdulrahman University Researchers Supporting Project number (PNURSP2023R39), Princess Nourah bint Abdulrahman University, Riyadh, Saudi Arabia.

## Conflict of interest

The authors declare that the research was conducted in the absence of any commercial or financial relationships that could be construed as a potential conflict of interest.

## Publisher’s note

All claims expressed in this article are solely those of the authors and do not necessarily represent those of their affiliated organizations, or those of the publisher, the editors and the reviewers. Any product that may be evaluated in this article, or claim that may be made by its manufacturer, is not guaranteed or endorsed by the publisher.

## Supplementary material

The Supplementary material for this article can be found online at: https://www.frontiersin.org/articles/10.3389/fmicb.2023.1175844/full#supplementary-material

## Footnotes

^1^ http://bioinformatics.psb.ugent.be/webtools/Venn/
