# Supplementary material for: Discovering common pathogenic processes between COVID-19 and HFRS by integrating RNA-seq differential expression analysis with machine learning
Source: Front Microbiol. 2023 May 5;14:1175844. doi: 10.3389/fmicb.2023.1175844 (PMC10208410; doi:10.3389/fmicb.2023.1175844)
Supplement: Supplementary file 1 [file Table_1.DOCX]

**Table S1:** Common 32 genes among HFRS and COVID-19

| **ENSEMBL IDs** | **Symbol** | **Gene name** |
| --- | --- | --- |
| ENSG00000051180 | RAD51 | RAD51 recombinase |
| ENSG00000111554 | MDM1 | Mdm1 nuclear protein |
| ENSG00000115738 | ID2 | inhibitor of DNA binding 2 |
| ENSG00000118707 | TGIF2 | TGFB induced factor homeobox 2 |
| ENSG00000129255 | MPDU1 | mannose-P-dolichol utilization defect 1 |
| ENSG00000138463 | SLC49A4 | solute carrier family 49 member 4 |
| ENSG00000139531 | SUOX | sulfite oxidase |
| ENSG00000149212 | SESN3 | sestrin 3 |
| ENSG00000154781 | CCDC174 | coiled-coil domain containing 174 |
| ENSG00000165092 | ALDH1A1 | aldehyde dehydrogenase 1 family member A1 |
| ENSG00000168137 | SETD5 | SET domain containing 5 |
| ENSG00000171055 | FEZ2 | fasciculation and elongation protein zeta 2 |
| ENSG00000221983 | UBA52 | ubiquitin A-52 residue ribosomal protein fusion product 1 |
| ENSG00000036257 | CUL3 | cullin 3 |
| ENSG00000099326 | MZF1 | myeloid zinc finger 1 |
| ENSG00000099860 | GADD45B | growth arrest and DNA damage inducible beta |
| ENSG00000105254 | TBCB | tubulin folding cofactor B |
| ENSG00000112701 | SENP6 | SUMO specific peptidase 6 |
| ENSG00000124762 | CDKN1A | cyclin dependent kinase inhibitor 1A |
| ENSG00000128585 | MKLN1 | muskelin 1 |
| ENSG00000129636 | ITFG1 | integrin alpha FG-GAP repeat containing 1 |
| ENSG00000145782 | ATG12 | autophagy related 12 |
| ENSG00000148926 | ADM | adrenomedullin |
| ENSG00000164919 | COX6C | cytochrome c oxidase subunit 6C |
| ENSG00000165494 | PCF11 | PCF11 cleavage and polyadenylation factor subunit |
| ENSG00000165682 | CLEC1B | C-type lectin domain family 1 member B |
| ENSG00000166228 | PCBD1 | pterin-4 alpha-carbinolamine dehydratase 1 |
| ENSG00000185973 | TMLHE | trimethyllysine hydroxylase, epsilon |
| ENSG00000197872 | CYRIA | CYFIP related Rac1 interactor A |
| ENSG00000198799 | LRIG2 | leucine rich repeats and immunoglobulin like domains 2 |
| ENSG00000258890 | CEP95 | centrosomal protein 95 |
| ENSG00000206199 | ANKUB1 | ankyrin repeat and ubiquitin domain containing 1 |

**Table S2:** Top 10 gene about from five different algorithms

| Degree | MCC | MNC | Closeness | Betweenness |
| --- | --- | --- | --- | --- |
| CDKN1A | CDKN1A | CDKN1A | CDKN1A | CDKN1A |
| RAD51 | RAD51 | CUL3 | RAD51 | RAD51 |
| CUL3 | CUL3 | RAD51 | CUL3 | GADD45B |
| UBA52 | UBA52 | ATG12 | GADD45B | ALDH1A1 |
| ALDH1A1 | GADD45B | UBA52 | UBA52 | CUL3 |
| GADD45B | ATG12 | GADD45B | ALDH1A1 | UBA52 |
| ATG12 | ALDH1A1 | SESN3 | SESN3 | SETD5 |
| SESN3 | SESN3 | TBCB | ATG12 | ADM |
| TBCB | TBCB | ALDH1A1 | MKLN1 | MKLN1 |
| SENP6 | SENP6 | SENP6 | TBCB | SENP6 |
